# In vitro dose-response of bromoform stabilized in vegetable oil on rumen fermentation and methane production

**DOI:** 10.3168/jdsc.2025-0988

**Published:** 2026-03-06

**Authors:** Lucas González-Chappe, Anaclara Daudet, Jeffrey L. Firkins, Alejandro E. Relling, Alejandro M. Pittaluga

**Affiliations:** Department of Animal Sciences, The Ohio State University, Wooster, OH 44691

## Abstract

Synthetically manufactured bromoform may provide a sustainable and economically viable alternative to macroalgae harvesting and cultivation for mitigating methane emissions in ruminants. Our objective was to evaluate the effect of varying doses of synthetic bromoform stabilized in vegetable oil on rumen fermentation, digestibility, and methane production using an in vitro batch culture system. Four treatments were evaluated: 0 (BR0), 1,000 (BR1), 2,500 (BR2), and 5,000 (BR5) mg bromoform/kg oil, added to batch culture vessels at a 2% DM basis. A randomized complete block design was applied across 3 periods, with incubations conducted for 3, 6, 12, 24, and 72 h in each period. Treatments were replicated 3 times at each incubation time. At 72 h, methane (mg/g of disappeared DM) was reduced by 32.3%, 96.4%, and 100% in BR1, BR2, and BR5, respectively, compared with BR0. The inclusion of bromoform at varying doses did not influence fermentation medium pH or apparent substrate disappearance, as measured by DM, NDF, and ADF disappearance. Bromoform inclusion modified the ruminal VFA profile. At 12 h, BR2 and BR5 treatments exhibited decreased acetate concentrations relative to BR1, whereas at 24 h, these treatments had reduced acetate concentrations compared with BR0. Moreover, by 72 h, BR5 had the lowest acetate concentrations among treatments. The acetate-to-propionate ratio was lesser in BR5 and marginally lesser in BR2 compared with BR0. Butyrate concentration was greater in BR1 and BR5 compared with BR0. At 72 h, BR2 and BR5 showed greater concentrations of valerate and caproate concomitant with a decrease in isobutyrate concentrations relative to BR0 and BR1. Isovalerate and 2-methylbutyrate concentrations were greater in BR1 compared with BR2 and BR5. The collective findings of this study indicate that synthetic bromoform inclusion (BR2 and BR5) markedly reduced methane production, preserved digestibility, and altered rumen fermentation characteristics, suggesting a compensatory shift in reducing equivalents utilization by the rumen microbiota.

## Highlights

• In vitro use of synthetic bromoform decreased methane production in batch cultures.
• In vitro use of synthetic bromoform does not decrease DM or NDF digestibility.
• In vitro use of synthetic bromoform affects VFA concentration in batch cultures.

## Graphical Abstract

**Figure:**
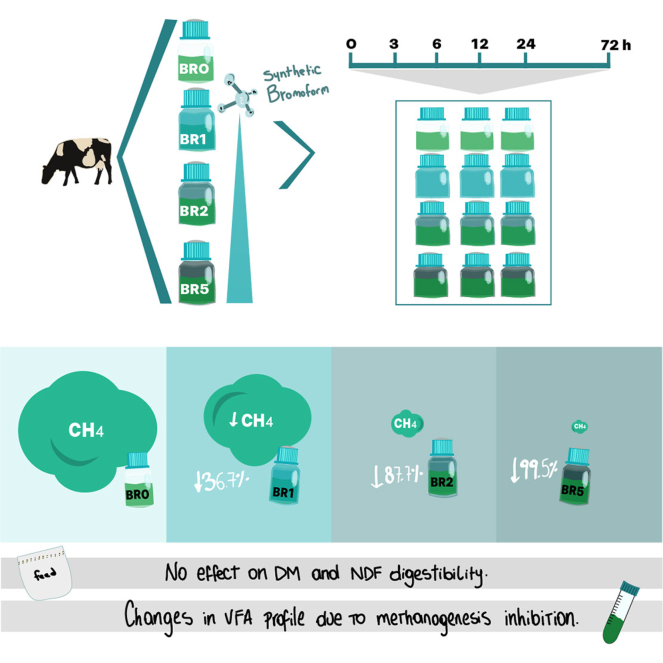
**Summary:**To evaluate the effect of increasing doses of bromoform on in vitro rumen fermentation and CH_4_ production, rumen fluid was incubated for up to 72 hours with 2% dry matter (DM) of vegetable oil alone (BR0) or oil containing increasing concentrations of bromoform (BR1, BR2, and BR5; 1,000, 2,500, and 5,000 mg/kg of oil, respectively). Increasing bromoform inclusion resulted in a dose-dependent reduction in CH_4_ production. Relative to BR0, CH_4_ production was reduced by approximately 32.3% with BR1, 96.4% with BR2, and 100% with BR5 at 72 hours of incubation. Despite the strong inhibition of methanogenesis, DM and neutral detergent fiber (NDF) digestibility were not affected by any treatment. In contrast, bromoform supplementation altered ruminal fermentation patterns, decreasing acetate concentrations, reducing the acetate-to-propionate ratio, and increasing proportions of more reduced volatile fatty acids (VFA). These changes are consistent with a redirection of metabolic hydrogen away from methanogenesis toward alternative fermentation pathways following methanogenic inhibition.

The macroalga *Asparagopsis taxiformis* (**AT**) has gained significant attention for its methane (CH_4_) mitigation properties in ruminants, attributed to the presence of halogenated compounds, particularly bromoform (CHBr_3_). In a dose-dependent response, seaweeds containing CHBr_3_ have been demonstrated to be potent anti-methanogenic feed additives both in vitro (Machado et al., 2016) and in vivo (Roque et al., 2019). Bromoform inhibits ruminal methanogenesis by disrupting cobamide-dependent methyl transfer reactions (Ungerfeld and Pitta, 2025).

Freeze-drying has been the preferred method for preserving volatile halogenated compounds in AT; however, there are challenges related to energy consumption and logistical limitations associated with freeze-drying whole seaweed biomass in large-scale processing (Vucko et al., 2017). Consequently, alternative technologies to preserve this volatile metabolite are required. A novel method consisting of homogenizing AT in vegetable oil successfully stabilized CHBr_3_ while maintaining efficacy (Cowley et al., 2024). Wild-harvested AT has been the main source thus far, yet sustainability and economic constraints dictate the need to develop cultivation methods in order to meet demand. However, potential ozone-depletion repercussions associated with AT farming, along with inefficient propagation techniques, may impede sufficient aquaculture production (Vijn et al., 2020). Therefore, synthetic CHBr_3_ could represent a viable strategy for CH_4_ mitigation under the current circumstances. Nevertheless, a significant knowledge gap exists regarding the dose-response relationship between synthetic CHBr_3_ and CH_4_ production. Building on the assumption that the combined effects of multiple CH_4_-reducing haloalkanes in AT biomass enhance the anti-methanogenic potency beyond that of CHBr_3_ alone (Ahmed and Nishida, 2024), this study aimed to evaluate varying doses of synthetic CHBr_3_ stabilized in vegetable oil on in vitro CH_4_ production, rumen fermentation, and DM, NDF, and ADF disappearance. We hypothesized that the greatest CHBr_3_ dose would maximize CH_4_ reduction without compromising rumen fermentation.

All experimental procedures involving animals were approved by the Institutional Animal Care and Use Committee of The Ohio State University (2017A00000069-R2). To evaluate the effects of varying doses of CHBr_3_ stabilized in vegetable oil, in vitro batch culture incubations were conducted in a randomized complete block design with 4 CHBr_3_-oil blends consisting of 0 (**BR0**), 1,000 (**BR1**), 2,500 (**BR2**), or 5,000 (**BR5**) mg CHBr_3_/kg soybean oil. The blends were prepared before the beginning of the experiment by thoroughly mixing liquid CHBr_3_ (96% purity, stabilized with 1%–3% ethanol) with the carrier (soybean) oil, and subsequently stored at 4 °C in Pyrex bottles, protected from ambient laboratory lighting. Rumen fluid was collected 2 h before the morning feed from 2 ruminally cannulated lactating Holstein cows housed indoors as inoculum donors. Cows were fed once daily for ad libitum intake of a TMR containing corn silage, alfalfa haylage, alfalfa hay, dry-rolled corn, soybean meal, soybean hulls, and a mineral premix (30%, 18%, 3%, 17.5%, 10.7%, 8.5%, and 12.3% on a DM basis, respectively), containing 16.3% CP, 28.5% NDF, and 17.9% ADF (DM basis).

Two liters of rumen fluid were collected from each cow by sampling multiple regions of the rumen using a rumen fluid extraction device (Rumen-Mate, Trans Agra International, Storm Lake, IA). In addition, 500 g of solid digesta were obtained from the floating mat. Both rumen fluid and solid digesta were immediately placed into prewarmed insulated containers and transported to the laboratory. Upon arrival, 1 L of rumen fluid and ∼125 g of ruminal solids from each cow were combined, homogenized for 1 min in a blender, and filtered through a double layer of cheesecloth while continuously flushing with CO_2_ to maintain anaerobic conditions. The rumen fluid blend was mixed with a preheated incubation medium (1:4 vol/vol) prepared according to Goering and Van Soest (1970) with minor modifications; the buffer contained 78.9 g/L sodium bicarbonate and 30 g/100 mL calcium chloride dihydrate. To ensure anaerobic conditions, 250 mL of reducing solution containing 40 mL/L of 1 *N* NaOH, 6.25 g/L of l-(+)-cysteine hydrochloride monohydrate, and 6.15 g/L of sodium sulfide nonahydrate was added.

Before rumen fluid collection, 600-mg samples of the fermentation substrate, ground at 2 mm, formulated to emulate the TMR offered to the donor cows, were preweighed into bags (25-μm porosity; Ankom Technology, Macedon, NY), heat sealed, and placed in 150-mL glass fermentation vessels. The rumen fluid–incubation medium solution (60 mL) was added to the fermentation vessels containing the substrate. Subsequently, the CHBr_3_-oil blends were added to the fermentation vessels at 2% of the substrate DM. Samples of the CHBr_3_-oil blends and the feed ingredients were collected before beginning the experiment to evaluate CHBr_3_ concentration and nutrient composition, respectively. Bromoform was quantified by gas chromatography–mass spectrometry at Bigelow Analytical Services (Portland, ME) following the procedure described by Posman et al. (2025), using hexane extraction with naphthalene as an internal standard. Samples of feed ingredients were dried in a forced-air oven (55°C for 72 h), ground using a Wiley mill with a 2-mm screen (Arthur H. Thomas Co., Philadelphia, PA), and sent to Rock River Laboratory (Wooster, OH) for analysis as described by Pittaluga et al. (2023).

The experiment was replicated across 3 independent periods (blocks). In each period, fermentation vessels were placed in triplicates into dedicated incubators with oscillation platforms at 39°C and 85 rpm for 3, 6, 12, 24, and 72 h. At each time point, 3 vessels per treatment were randomly selected for incubation termination by immersion in ice following headspace gas pressure measurement. Additionally, before initiating incubations (0 h), 1 vessel per treatment was randomly selected for determination of baseline rumen VFA as well as the substrate's NDF and ADF concentrations.

Immediately after stopping fermentation, headspace gas pressure was measured using a pressure transducer (Traceable Model 3463, Cole-Parmer, Vernon Hills, IL). Gas pressure was converted to volume using a linear regression obtained from triplicate vessels calibrated with known gas volumes on each experimental day. Headspace gas samples were subsequently collected into pre-evacuated 7-mL vials (Exetainer vials, Labco Ltd., Lampeter, UK) for CH_4_ and CO_2_ quantification. Upon collection of gas samples, pH of the fermentation medium was measured using a portable pH meter (Environmental Express model P200-02, Cole-Palmer, Vernon Hills, IL), and a 5-mL sample of the fermentation medium from each vessel was preserved with 1 mL of 25% metaphosphoric acid and stored at −20°C for further VFA analyses.

Bags containing residual substrate were removed from fermentation vessels, rinsed with cold water until the effluent was clear, and dried at 55°C for 72 h in a forced-air oven before analyzing the apparent DM, NDF, and ADF digestibilities. Dried bags were weighed, and the DM content of the residual substrate was calculated. Bags were then analyzed for NDF and ADF content as described by Pittaluga et al. (2023). The NDF disappearance was calculated as 100 − 100 × (final amount of NDF ÷ initial amount of NDF), and ADF disappearance was calculated using ADF amounts.

Headspace gas samples (0.5 mL) were analyzed for CH_4_ and CO_2_ concentrations using GC. Samples were manually injected into the GC inlet using a gas-tight syringe. The GC system was equipped with a J&W 113-3133 capillary column (30 m × 320 μm i.d. × 3.00 μm GS-Carbonplot film; Agilent Technologies, Santa Clara, CA); helium was used as the carrier gas. The oven temperature was maintained at 35°C, with injector and detector temperatures set at 175°C and 180°C, respectively. Methane was detected using a thermal conductivity detector. Quantification was based on peak area, which was automatically integrated using Chromperfect software (version 10, Justice Laboratory Software, Denville, NJ).

Acidified aliquots of the fermentation medium were analyzed for VFA by GC, according to the procedure described by Roman-Garcia et al. (2021), using a 195°C oven temperature set. The moles of pairs of reducing equivalents (**[2H]**) released or consumed per mole of end product were estimated using the stoichiometric approach described by Guyader et al. (2017) for acetate, propionate, butyrate, valerate, and caproate.

Data were analyzed using the GLIMMIX procedure of SAS (9.4; SAS Institute Inc., Cary, NC). Data distribution and outliers were assessed using the UNIVARIATE procedure. The statistical model included treatment, time (hours after inoculation), and their interaction as fixed effects; block was included as a random effect. Post-hoc comparisons were performed using the Tukey–Kramer test at each particular time when a treatment × time interaction (*P* ≤ 0.05) occurred. Least squares means were considered different when *P* ≤ 0.05 and marginally different when 0.05 < *P* ≤ 0.10. Polynomial contrasts were used to evaluate linear and quadratic dose-response effects of dietary CHBr_3_ on response variables in which no treatment × time interaction was detected.

The concentration of CHBr_3_ was below the limit of detection for BR0 and was 1,060, 2,710, and 5,330 mg/kg of oil for BR1, BR2, and BR5, respectively. Total gas production linearly decreased (*P* < 0.01) as CHBr_3_ increased. A treatment × time interaction was observed for CH_4_ production (*P* ≤ 0.03; Figure 1). At 3 h, CH_4_ production was lesser in BR1, BR2, and BR5 compared with BR0. At 6 h, CH_4_ production was lesser in BR2 and BR5 compared with BR1 and BR0. At 12, 24, and 72 h, CH_4_ production was lesser in BR1 than BR0; BR2 and BR5 were also lesser compared with both BR0 and BR1. Although anti-methanogenic efficacy evidence of synthetic haloalkanes exists in the literature (Thorsteinsson and Nielsen, 2025), only a few experiments have assessed the effectiveness of synthetic CHBr_3_ in reducing CH_4_ emissions (Ahmed and Nishida, 2024; Kelly et al., 2025).

**Figure 1:**
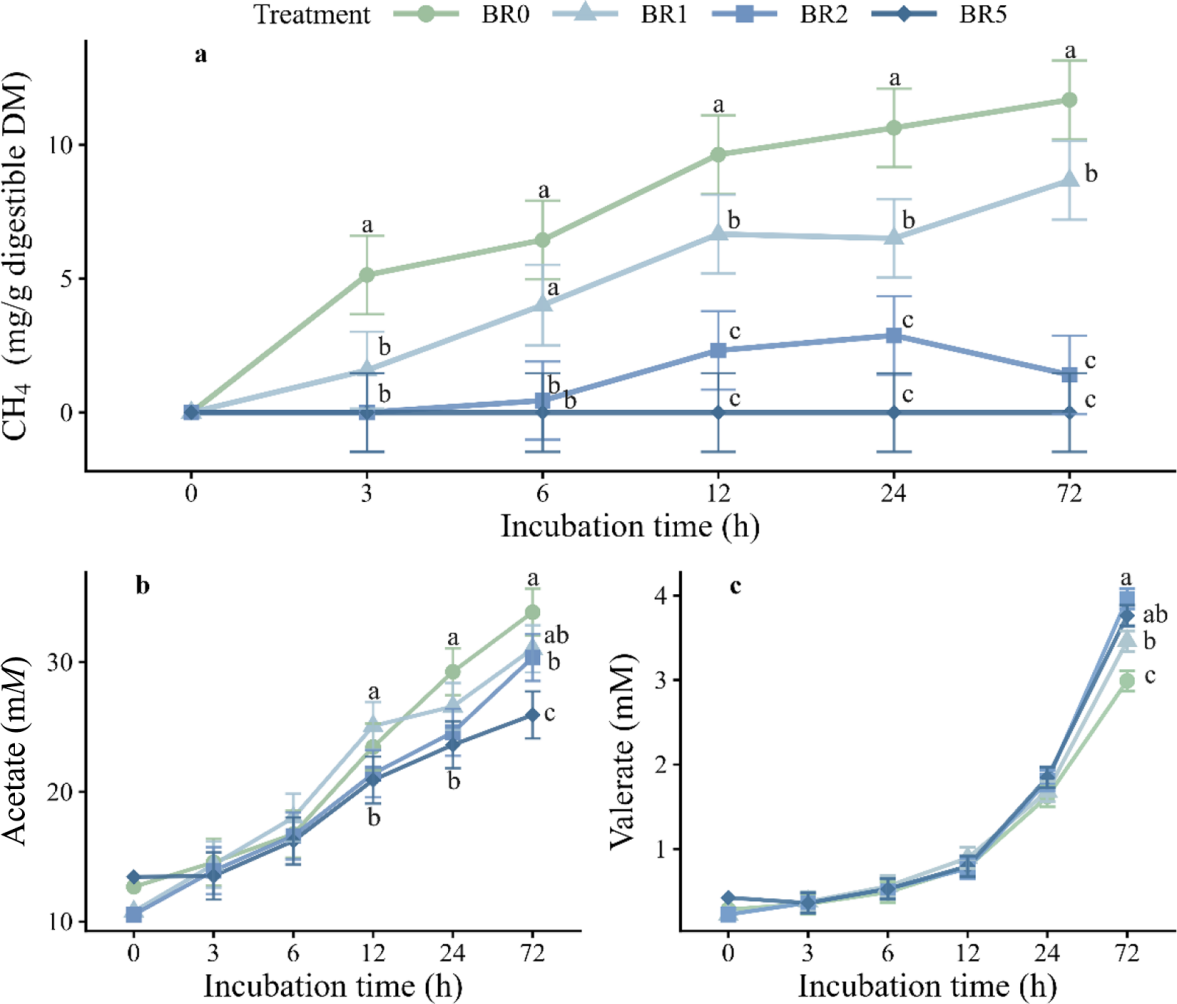
Methane (CH_4_; mg/g digestible DM; panel a), acetate (m*M*; panel b), and valerate (m*M*; panel c) during in vitro incubation for the control group supplemented with oil at 2% of DM without bromoform (BR0), and treatments supplemented with the blend at 2% of DM with 1,000 (BR1), 2,500 (BR2), and 5,000 (BR5) milligrams of bromoform per kilogram of vegetable oil. Different lowercase letters (a–c) within a panel indicate differences between treatments at the same time point (*P* < 0.05). Error bars represent SEM.

Kelly et al. (2025) evaluated the effects of an oil-based or a powdered formulation containing synthetic CHBr_3_ on in vivo CH_4_ emissions in feedlot cattle and observed a 95% decrease in CH_4_ production when the oil-based formulation was added to the finishing diet. On average, in our experiment, CH_4_ was reduced by 36.7%, 87.7%, and 99.5% for BR1, BR2, and BR5, respectively. The CHBr_3_ inclusion rate in the oil-based formulation used by Kelly et al. (2025) was 32.2 mg/kg of DM intake (equivalent to 0.032 mg/g of feed), which falls between the supplementation levels of our BR1 (0.02 mg/g of substrate) and BR2 (0.05 mg/g of substrate) treatments. Nevertheless, the reduction in CH_4_ production observed in our experiment at similar doses (BR1 and BR2) was smaller in magnitude.

The divergent CH_4_ mitigation efficacies achieved between our experiment and that of Kelly et al. (2025) might be related to the composition of the basal diet. In the prior study, animals were fed a corn grain-based diet, which differs from the forage-based substrate used in the present experiment. Consistent with differences in CH_4_ production due to the basal diet, Sun et al. (2025) reported reduced antimethanogenic efficacy of synthetic CHBr_3_ in cows consuming fresh pasture diets. Using synthetic CHBr_3_ with a forage-based substrate, Stepanchenko et al. (2025) reported a 98% reduction in CH_4_ production in vitro; however, the single dose applied in their study was more than 10-fold greater (∼1.5 mg CHBr_3_/g substrate) than our BR5 treatment (0.10 mg CHBr_3_/g substrate). Roque et al. (2021) reported that the extent of CH_4_ reduction with AT was inversely correlated with the forage and NDF concentrations of the diet. The authors attributed this pattern to a reduced abundance of methyl-coenzyme M reductase in low-forage environments, thereby increasing the susceptibility of methanogens to antimethanogenic compounds targeting this enzyme. Alternatively, Ungerfeld and Pitta (2025) proposed a shift in methanogens population toward archaeal communities less susceptible to haloalkanes.

*Asparagopsis taxiformis* supplementation has shown greater antimethanogenic efficacy than CHBr_3_ alone (Ahmed and Nishida, 2024), suggesting that greater doses of synthetic CHBr_3_ are typically required to achieve similar reductions. Romero et al. (2023) reported a 98% decrease in CH_4_ production in vitro using AT, with a CHBr_3_ dose of 0.012 mg/g DM, whereas Stepanchenko et al. (2025) achieved similar reductions using 1.1 mg of synthetic CHBr_3_/g DM. Although these doses were ∼1.2 and 10 times greater, respectively, than the greatest CHBr_3_ dose used in our experiment, comparable decreases in CH_4_ production were observed. These findings support the notion that lesser CHBr_3_ doses may be sufficient to achieve near-maximal CH_4_ mitigation under certain conditions.

We did not observe differences in apparent DM, NDF, and ADF disappearances (*P* ≥ 0.26; Table 1). Inhibition of methanogenesis has generally not impaired in vitro (Ungerfeld and Pitta, 2025) or in vivo (Ungerfeld, 2018) feed digestibility. However, Machado et al. (2016) reported that elevated inclusion rates of AT (>10% of dietary OM) reduced feed digestibility, and concluded that digestion impairments could be related to the potential toxicity of halogenated compounds on rumen microbial populations and not a consequence of methanogenesis inhibition.

**Table 1:** In vitro gas production, feed degradability (%), and VFA production for the control group supplemented with oil at 2% of DM without bromoform (BR0), and treatments (T) supplemented with the blend at 2% of DM with 1,000 (BR1), 2,500 (BR2), and 5,000 (BR5) milligrams of bromoform per kilogram of oil

Item	Treatment				SEM	*P*-value		
	BR0	BR1	BR2	BR5		T	Time (t)	T × t
Gas production								
Total gas^1^	234	243	219	210	21.7	0.04	<0.01	0.83
Methane^2^	7.29	4.61	0.90	0.04	1.23	<0.01	<0.01	<0.01
Carbon dioxide^2^	97.6^ab^	103^a^	89.3^b^	97.1^ab^	3.38	0.05	<0.01	0.09
Disappearance (%)								
Dry matter (% DM)	34.7	35.2	34.9	34.2	0.98	0.35	<0.01	0.94
NDF (% NDF)	19.5	17.9	19.6	18.7	1.11	0.52	<0.01	0.93
ADF (% ADF)	20.0	17.6	20.1	17.9	1.83	0.26	<0.01	0.83
VFA concentration (m*M*)								
Total VFA^3^	40.3	39.4	38.4	37.7	3.42	0.26	<0.01	0.46
Acetate	21.8	21.0	19.6	18.9	1.57	<0.01	<0.01	0.02
Propionate^3^	10.5	10.3	10.1	9.79	0.72	0.25	<0.01	0.12
Butyrate^4^	5.49	6.08	5.91	6.21	0.28	0.01	<0.01	0.42
Valerate	1.09	1.20	1.28	1.29	0.11	0.01	0.02	<0.01
Isobutyrate	0.40	0.44	0.39	0.38	0.02	0.01	<0.01	0.02
Isovalerate^5^	0.37	0.41	0.37	0.37	0.02	0.02	<0.01	0.06
2-Methylbutyrate^6^	0.41	0.45	0.40	0.40	0.02	0.01	<0.01	0.14
Caproate	0.32	0.38	0.41	0.35	0.04	0.01	<0.01	<0.01
Acetate:propionate ratio^7^	2.35	2.32	2.26	2.18	0.03	<0.01	<0.01	0.21
Metabolic hydrogen production ([H]; mmol)								
[H] consumed	0.49	0.50	0.50	0.47	0.05	<0.53	<0.01	0.01
[H] produced	1.56^a^	1.54^ab^	1.35^ab^	1.31^b^	0.21	0.02	<0.01	0.08
[H] balance	1.07	1.04	0.85	0.84	0.11	0.01	<0.01	0.03

a,b Different superscripts within a row represent a mean separation at *P* < 0.05.

1 In production: mL/g of digestible DM. Linear effect *P* < 0.01, quadratic and cubic effects *P* ≥ 0.16.

2 mg/g of digestible DM.

3 Linear effect *P* = 0.05, quadratic and cubic effects *P* ≥ 0.78.

4 Linear effect *P* = 0.05, quadratic effect *P* = 0.34, cubic effect *P* = 0.05.

5 Linear effect *P* = 0.23, quadratic effect *P* = 0.32, cubic effect *P* < 0.01.

6 Linear effect *P* = 0.03, quadratic effect *P* = 0.75, cubic effect *P* < 0.01.

7 Linear effect *P* < 0.01, quadratic and cubic effects *P* ≥ 0.92.

No treatment effect or treatment × time interaction was observed for total VFA (**tVFA**) concentration (*P* = 0.46; Table 1), but a linear decrease (*P* = 0.05) of tVFA was observed when CHBr_3_ increased. This decrease may be attributed to a decrease in acetate and propionate concentrations. A treatment × time interaction was observed for acetate (*P* = 0.02; Table 1). Acetate concentrations did not differ between treatments at 0, 3, or 6 h (*P* ≥ 0.63; Figure 1); however, at 12 h, BR5 had a lesser concentration (*P* < 0.03) and BR2 showed marginally lesser concentration (*P* < 0.07) compared with BR1. At 24 h, both BR2 and BR5 showed lesser acetate concentrations than BR0, and, at 72 h, BR5 had the least acetate concentration between treatments (*P* ≤ 0.01).

Propionate did not have a treatment × time interaction (*P* = 0.12; Table 1). However, a linear decrease (*P* = 0.05) of propionate concentration occurred as CHBr_3_ increased. Despite both acetate and propionate concentration decreasing as CHBr_3_ increased, the acetate:propionate ratio decreased (*P* linear <0.01; quadratic and cubic ≥0.92) with CHBr_3_ supplementation. An increase (*P* linear = 0.05, quadratic = 0.75, and cubic = 0.05) for butyrate concentration was observed with the increase of CHBr_3_. A time × treatment interaction was observed for valerate concentration (*P* ≤ 0.01; Table 1). At 72 h, valerate concentrations were greater in BR1, BR2, and BR5 compared with BR0, and BR2 had a greater valerate concentration than BR1.

A time × treatment interaction was observed for isobutyrate and caproate concentrations (*P* ≤ 0.02, Table 1). At 12 h, isobutyrate concentration was lesser in BR2 and BR5 compared with BR1. At 24 h, isobutyrate concentration was marginally lesser in BR5 compared with BR1. At 72 h, BR5 had the least isobutyrate concentrations among treatments, and BR2 had lesser concentration compared with BR1. For caproate, at 12 h, BR1 had a marginally greater concentration compared with BR2, and BR1 had greater concentration compared with BR5. At 24 h, both BR0 and BR1 had greater caproate concentration than BR5. However, at 72 h, BR2 had the greatest concentrations among treatments, whereas BR5 had greater caproate concentration than BR0 and BR1. Additionally, BR1 had greater caproate concentration than BR0. A cubic effect was observed for isovalerate and 2-methylbutyrate concentrations, with BR1 showing the greatest isovalerate concentration between treatments (*P* ≤ 0.05).

A time × treatment interaction was observed for [2H] consumed (*P* ≤ 0.02). No differences were observed up to 12 h. At 24 h, BR5 showed less [2H] consumption than BR0. At 72 h, BR2 had greater [2H] consumption than the other treatments. A marginal treatment effect was observed for [2H] produced, with BR5 producing less [2H] compared with BR0 (*P* = 0.06). A time × treatment interaction was observed for [2H] balance (*P* ≤ 0.03). At 24 h, marginal reductions in [2H] balance were observed in BR2 and BR5 compared with BR0 (*P* ≤ 0.09). At 72 h, [2H] balance was lesser in BR5 compared with BR0 and BR1, and [2H] balance was lesser in BR2 than in BR0.

Previous studies have reported reductions in tVFA when methanogenesis was inhibited with AT in vitro (Ahmed et al., 2023; Terry et al., 2023). However, in the research conducted by Terry et al. (2023), these reductions were accompanied by temporary decreases in OM or NDF degradability. Ahmed et al. (2023) reported a 2.7% reduction in tVFA at a 2.5% AT inclusion level in vitro. Similarly, Kinley et al. (2016) observed decreases in tVFA when AT was included at concentrations above 2%. Both studies also documented increases in the molar production of propionate and butyrate, together with reductions in acetate, suggesting that the decline in tVFA may reflect a lesser total number of VFA moles resulting from shifts in VFA molar proportions. Most in vivo studies have reported either no effect (Nolan et al., 2010; Martínez-Fernández et al., 2014; Zhang et al., 2021) or only modest decreases in tVFA (Kelly et al., 2025) under methanogenesis inhibition.

When methanogenesis is inhibited, the primary sink for [2H] in the rumen is disrupted. Consequently, [2H] accumulates, increasing the partial pressure of hydrogen and hindering the reoxidation of reduced cofactors (Ungerfeld, 2020). As a result, microbial populations redirect [2H] toward alternative fermentation pathways, leading to an increased production of more reduced end products (Ungerfeld, 2020; Ungerfeld and Pitta, 2025). The lesser concentration of acetate under CHBr_3_ supplementation is consistent with its role as a net [2H]-generating pathway in the rumen (Firkins and Mitchell, 2023). Acetate is the predominant VFA in the rumen; however, when methanogenesis is inhibited, the formation of more reduced VFA allows microorganisms to redirect excess [2H] toward alternative electron sinks (Ungerfeld, 2020). Nevertheless, the lesser [2H] capture evidenced in BR5 suggests that microorganisms under this treatment may not be able to reroute [2H] toward the synthesis of more reduced fermentation products.

Propionate synthesis, typically a prevalent [2H]-consuming pathway, was limited, as evidenced by the observed acetate:propionate decrease being driven primarily by reduced acetate production rather than by enhanced propionate formation. This implies that metabolic or microbial limitations impeded [2H] redirection toward the succinate or acrylate pathways (Ungerfeld and Pitta, 2025). Simultaneously, the increases in butyrate, valerate (derived from propionate), and caproate production observed herein, might indicate that these fatty acids served as alternative [2H] sinks (Jeong et al., 2024). The thermodynamic advantage of [2H] disposal via elongation of VFA carbon chains in a methane-inhibited rumen environment has been previously reported in vivo and in vitro (Machado et al., 2016).

Methane production represents an energy loss of up to 12% of the gross energy intake (Johnson and Johnson, 1995). Thus, inhibition of methanogenesis could theoretically redirect the conserved energy toward VFA synthesis, the primary energy source for ruminants. However, similar to our findings, previous authors using CH_4_ inhibitors did not observe greater tVFA concentration or production (Cowley et al., 2024; Kelly et al., 2025). Nevertheless, shifts in VFA profiles may alter tVFA concentration without necessarily changing the total amount of carbon or [2H] conserved by microorganisms. However, based on the [2H] balance calculated in the current experiment, it can be surmised that when CH_4_ production is substantially inhibited, the spared dietary energy was not effectively rerouted toward anabolic processes. Instead, the observed decrease in H-generating pathways implies a metabolic recalibration, wherein the rumen microbiota adapted to the altered redox landscape by downregulating reactions that generate [2H]. A detailed assessment of metabolic hydrogen dynamics was beyond the scope of this study, and we acknowledge the limitations of the balance approach (i.e., the absence of measurements of gaseous and dissolved H_2_). Instead, our objective was to perform a molar balance of [2H] released or consumed per mole of fermentation end products. Despite such limitations, the estimates may still provide useful mechanistic insight for future in vivo research.

The collective findings of this study indicate that synthetic CHBr_3_ inclusion (BR2 and BR5) markedly reduced CH_4_ production, preserved digestibility, and altered rumen fermentation characteristics, suggesting a compensatory shift in reducing equivalents utilization by the rumen microbiota characterized by a downregulation of H-generating pathways. Additional research is required to determine the optimal CHBr_3_ dose in vivo, with the present doses serving as reference.
